# Measuring the Effective Electro-Optic Coefficient of Low-Temperature-Prepared Lead Zirconate Titanate Thin Films

**DOI:** 10.3390/ma18040837

**Published:** 2025-02-14

**Authors:** Bin Li, Hongyan Yu, Chen Yang, Jungan Wang, Yu Han, Feng Qiu

**Affiliations:** 1 Hangzhou Institute for Advanced Study, University of Chinese Academy of Sciences, No. 1 Xiangshanzhi Lane, Hangzhou 310024, China; libin@ucas.ac.cn (B.L.);; 2 Shanghai Institute of Technical Physics, Chinese Academy of Sciences, Shanghai 200083, China; 3 Juhe Electro-Optic (Hangzhou) Tech. Co., Ltd., Hangzhou 310024, China

**Keywords:** lead zirconate titanate film, low temperature, CMOS compatibility, electro-optic coefficient

## Abstract

Developing lead zirconate titanate (PZT)-based electro-optic (EO) modulators is vital for integrated photonics. The high annealing temperature required for the processing of PZT thin films restricts their compatibility with modern complementary metal–oxide–semiconductor (CMOS) technology. In this work, high-quality PZT films were fabricated on SiO_2_/Si substrates at a low annealing temperature of 450 °C. The PZT films demonstrated a preferential (100) orientation and were uniform and crack-free. Based on the low-temperature PZT films, we subsequently designed and fabricated a Mach–Zehnder Interferometer (MZI) waveguide modulator. The measured half-wave voltage (*V_π_*) was 4.8 V at a wavelength of 1550 nm, corresponding to an in-device EO coefficient as high as 66 pm/V, which shows potential use in optical devices. The results reported in this work show great promise for the integration of PZT thin films with other complex systems.

## 1. Introduction

EO materials play a unique and crucial role in integrated photonics [1,2,3]. Despite the prevalence of LiNbO_3_ thin films in commercially established EO modulators, driven by their high-speed response and low optical loss [4,5], complex processing and high cost are driving people to pursue better EO properties and miniaturization and to explore alternative materials. PZT thin films are highly promising as an EO material for modulator fabrication, owing to their outstanding EO effect [6,7,8,9]. Nevertheless, the high annealing temperature processing of PZT thin films presents a barrier to the direct integration of micro-electromechanical systems (MEMS) devices onto CMOS devices. To date, there has been limited research on the exploration of low-temperature PZT waveguide modulators. There is an urgent need to investigate the properties of EO modulators based on low-temperature PZT.

To the best of our knowledge, conventional processing of PZT thin films typically requires annealing temperatures exceeding 600 °C to crystallize the material into the desired perovskite phase, which is known for exhibiting outstanding EO properties [10,11,12,13,14]. For instance, Zhu et al. [10] demonstrated the preparation of PZT thin films on Corning glass substrates using the magnetron sputtering technique at a high annealing temperature of 650 °C. Their study revealed the attainment of an EO coefficient of 185.3 pm/V. Qiu’s work investigated highly (100)-oriented PZT thin films deposited on SiO_2_/Si and demonstrated a large in-device EO coefficient of 133 pm/V [15]. Beeckman et al. demonstrated the trade-offs for the design of a Mach–Zehnder modulator in a hybrid PZT/Si device for a TE optical mode, achieving bandwidths beyond 60 GHz via a Mach–Zehnder modulator with *V_π_* = 7.0 V [16]. However, the annealing temperature required for these processes significantly exceeds the maximum thermal budget compatible with CMOS devices, typically around 450 °C [17].

To overcome this limitation, considerable research efforts have been devoted to the development of low-temperature processing techniques [18,19]. However, limited research has been conducted on the exploration of low-temperature PZT waveguide modulators. As one of the most effective approaches, the development of low-temperature sol–gel-processed PZT films has been ongoing for a long time, with only a few successful outcomes achieved. Peng et al. [20] reported the fabrication of PZT thin films using the sol–gel method at an ultra-low temperature of approximately 450 °C and demonstrated a large spontaneous polarization of approximately 30 μC/cm^2^ and a high dielectric breakdown strength of approximately 2900 kV/cm. Takamura et al. [21] reported the fabrication of PZT films with large remanent polarization and a small leakage current at approximately 450 °C using a solution combustion synthesis method. Nevertheless, the orientation of the resulting PZT thin films could not be controlled, and the utilization of Pt as the bottom electrode made them incompatible with photonic technology due to high optical loss [22]. A straightforward fabrication process capable of producing PZT thin films with feasible orientation control at low temperatures for EO modulators is highly desired.

In this work, highly (100)-oriented PZT films have been fabricated on SiO_2_/Si substrates at low annealing temperatures (450 °C) by utilizing the spin-coating method. To further explore the potential for use in optical devices, we fabricated a low-temperature PZT MZI waveguide modulator. The performance of the modulator was also demonstrated.

## 2. Experimental Methods

The preparation of the PZT thin films was as follows: The substrate was a commercial Si wafer with a 3 μm thick thermal oxide SiO_2_ layer. To achieve pure perovskite-structured PZT thin films, an atomic seed layer [15] was deposited on the surface of the wafer, which facilitates the crystallization of PZT in the desired atomic lattice orientation. The precursor solution for the seed layer was commercially lanthanum-based solvent [purchased from Juhe Electro-Optic (Hangzhou) Tech. Co. Ltd., Hangzhou, China]. Before spin-coating, the SiO_2_/Si substrates were rinsed successively in acetone, ethanol, and de-ionized water and then dried in a vacuum drying oven. Subsequently, the cleaned wafer underwent oxygen plasma treatment. Afterward, the seed layers were spin-coated onto the substrates and subjected to heat treatment on a hot plate at 440 °C for 2 to 5 min.

The PZT precursor solutions were prepared using a method based on a previously reported procedure [21], with minor modifications. Specifically, the Pb sol was prepared by dissolving lead nitrate, ammonium nitrate, and tricine in a 1:1:1 molar ratio in 2-methoxyethanol, with the solution being stirred at 110 °C for 2 h to form a transparent, homogeneous solution. The Zr sol was prepared by dissolving zirconyl nitrate, ammonium nitrate, and urea in 2-methoxyethanol under a nitrogen atmosphere (N_2_), while the Ti sol was obtained by dissolving titanium tetraisopropanolate, ammonium nitrate, and urea in 2-methoxyethanol under N_2_, following a similar procedure for both. The Zr-Ti sol was then prepared by mixing the Zr sol and Ti sol at room temperature. Finally, the Pb sol was added to the Zr-Ti sol while stirring for 0.5 h, resulting in a stable PZT sol. The PZT precursor solution is spin-coated onto the seed layers and then dried under vacuum conditions, followed by pyrolysis at 250 °C for 20 min on a hot plate to remove the organic components. The heating and cooling ramp rates for the specimen were both set to 10 °C/min. Then, the amorphous PZT thin films were annealed by using a rapid thermal annealing system at preset temperatures under an oxygen atmosphere. Each spin-coating, pyrolysis, and annealing step yielded a layer of approximately 100 nm thickness; thus, the process cycle was repeated twice to achieve a film thickness of 200 nm.

Next, the MZI waveguide was fabricated by using photolithography and inductively coupled plasma (ICP) etching. The top cladding layer, fabricated using the spin-coating process, was deposited on the PZT film, followed by heat treatment on a hot plate at 150 °C for 2.0 h to form a 200 ± 10 nm thick film. The co-planar Al electrodes were prepared using a thermal evaporation and lift-off process.

The crystallographic structures of the PZT thin films were analyzed by X-ray diffractometry (XRD, Bruker D8 Advance, Bremen, Germany) using CuKα radiation. The cross-section and surface morphology of the films were measured by field emission scanning electron microscopy (SEM, JEOL 6335F, Tokyo, Japan) and atomic force microscopy (AFM) analysis. The chemical states of the constituent elements in the PZT thin films were determined using an X-ray energy dispersive spectroscope (EDS).

## 3. Results and Discussion

### 3.1. PZT Film Characterization

**Figure 1:**
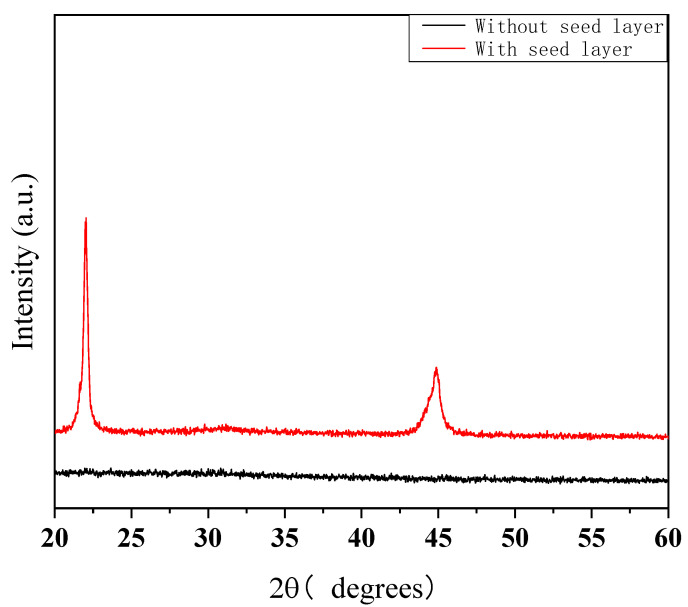
XRD patterns of the PZT thin films, with and without a seed layer, were obtained after annealing at 450 °C for 1.0 h.

**Figure 2:**
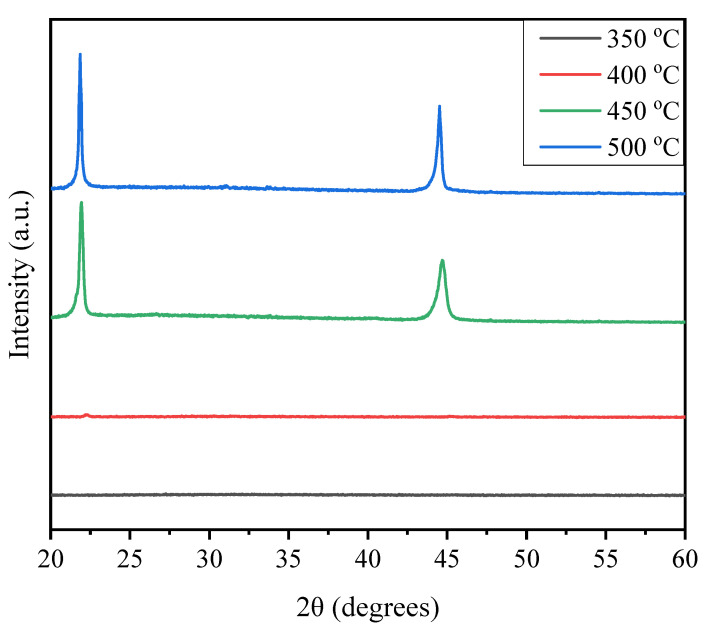
XRD patterns of the PZT thin films annealed at different temperatures.

Figure 1 and Figure 2 show the XRD patterns of the PZT thin films obtained via the spin-coating method, with diffractograms recorded for 2θ angles ranging from 20° to 60°. As depicted in Figure 1, it shows the XRD patterns of the PZT thin film deposited on SiO_2_/Si substrates and annealed at 450 °C for 1.0 h with or without a seed layer. As can be seen, the PZT thin film with a seed layer was well crystallized and exhibited a highly consistent (100) orientation, whereas the PZT film without a seed layer was not crystallized. As expected, introducing the seed layer can effectively promote the formation of a perovskite-structure. It has been reported [9] that the La_2_O_2_CO_3_ layer is formed after heat treatment of the seed layer at 440 °C in ambient air. This process ensures the creation of a stable and well-defined seed layer that promotes the nucleation of the PZT film. The lattice mismatch between the PZT film and La_2_O_2_CO_3_ is very small, with theoretical lattice constants of 4.055 Å for PZT and 4.076 Å for La_2_O_2_CO_3_, resulting in a lattice mismatch of only 0.505%. This small mismatch indicates a good lattice match between the PZT film and the La_2_O_2_CO_3_ seed layer, which helps in achieving high-quality crystallization and optimal electro-optic performance. These results indicate that the formation of the intermediate phase reduces the nucleation activation energy, thereby enabling the production of the perovskite phase at a lower temperature.

Figure 2 shows the phase evolution of the PZT thin films after annealing at different temperatures with the seed layers under an oxygen atmosphere. All the PZT films shown in Figure 2 were deposited identically and then post-annealed separately at 350, 400, 450, and 500 °C for 1.0 h. It can be observed that the PZT films were amorphous when annealed below 450 °C and well crystallized to the perovskite phase after being annealed at 450 °C. As the annealing temperature increased to 500 °C, no other impurity phase was detected, indicating that the phase transformation process had been accomplished. Moreover, no pyrochlore phase was found in the PZT films, and a pure perovskite structure was observed, sharing the same preferred orientation along the (100) direction as the seed layers.

**Figure 3:**
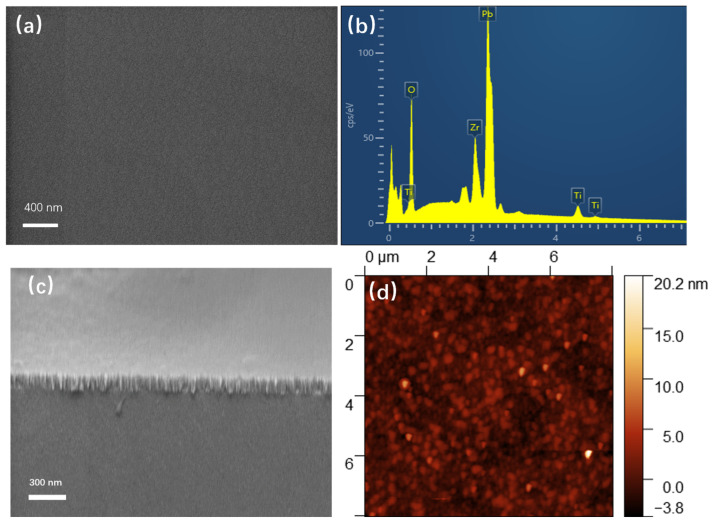
(**a**) Surface morphology of a PZT film observed by SEM. (**b**) EDS of the PZT thin film. (**c**) Cross-sectional SEM image. (**d**) AFM topographic image.

To further investigate the quality of PZT films annealed at 450 °C, we demonstrated the elemental content and surface morphology. Ensuring precise control over thin-film stoichiometry, especially the Pb/Zr/Ti ratio, emerged as a crucial concern in this work, as it directly influenced the EO properties of the PZT thin films. The EDS of the PZT thin films, as illustrated in Figure 3b, confirms the presence of Pb, Zr, Ti, and O. These elements collectively account for the composition of perovskite-structured PZT thin films. Semi-quantitative analysis further reveals that the content ratio of Ti to Zr is approximately 0.91, indicating proximity to the morphotropic phase boundary (MPB) compositions that are known for their excellent electro-optic properties [10]. Figure 3c shows a cross-sectional SEM image of the PZT film, illustrating that the film was well crystallized and crack-free and exhibited a uniform thickness of approximately 190 nm. The surface had a relatively smooth profile, as shown in Figure 3d, indicating a grain size of approximately 30 nm and a surface roughness of about 1.2 nm. The small grain size and surface roughness contributed to reducing propagation losses, leading to low light-scattering losses. Furthermore, the favorable stoichiometry and homogeneity of the films effectively minimized propagation losses, thereby enhancing the practicality of the device.

### 3.2. Fabrication of PZT Waveguide Modulators

**Figure 4:**
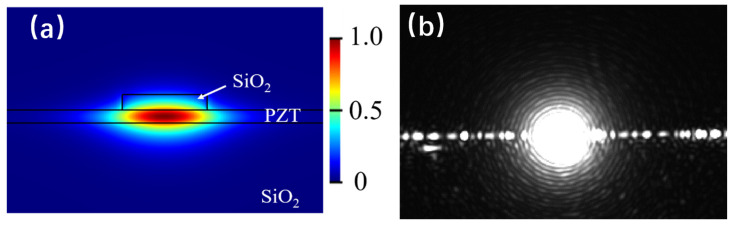
(**a**) Simulated optical model profile in the waveguide modulator; (**b**) TE mode output light collected by a CCD camera.

Given the demonstrated high crystalline quality of PZT thin films processed at a low temperature (450 °C), excellent electro-optic effects were expected to be obtained in modulators. An MZI waveguide modulator based on the film was fabricated. According to our design, the thickness of the PZT film was 200 ± 10 nm, and the top cladding layer had a width of 2.0 µm and a thickness of 200 ± 10 nm. The top cladding layer was made of SiO_2_, and the preparation method involved spin-coating a sol–gel SiO_2_ layer onto the PZT film, followed by a two-step baking process in air: first at 120 °C for 2 h, then at 150 °C for another 2 h. The co-planar electrodes were fabricated on top of the PZT layer, each consisting of a 200 nm thick aluminum layer, with a distance of 6.0 µm between them. Figure 4a illustrates the fundamental TE mode within the waveguide, simulated using Lumerical’s optical mode calculation. In the simulation, the refractive indices were 2.40 for the PZT film and 1.44 for the SiO_2_ layer at a wavelength of 1550 nm [23]. It can be seen that the optical fields were confined within the PZT thin film, with a 42% confinement factor.

**Figure 5:**
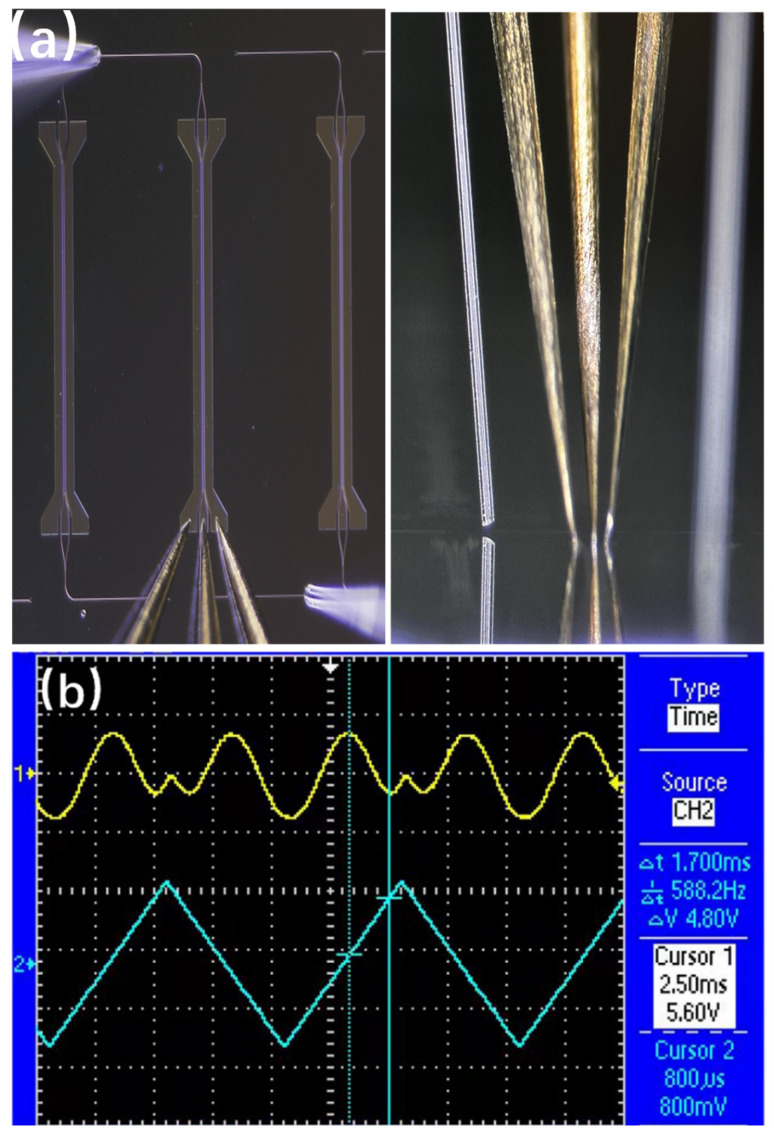
(**a**) Photograph of the low-temperature PZT-based MZI modulator, showing the position and layout of the electrical contacts. (**b**) The half-wave voltage (Vπ) with a 1 KHz triangular signal is shown; the characteristic voltage of the PZT-based MZI modulator for this power is 4.8 V at 1550 nm.

Based on the design outlined above, the PZT MZI waveguide modulator was fabricated, as illustrated in Figure 5a. A detailed schematic diagram of the interferometer has been included, which clearly illustrates the waveguide pattern, as well as the position and layout of the electrical contacts. Subsequently, the device underwent measurement using an end-face coupling system. The input light from the laser, operating at a wavelength of 1550 nm, was coupled into the device through a polarization-maintaining fiber. Figure 4b shows the output light captured by the CCD when the input light was polarized in the TE direction. A distinct single-mode pattern was observed, indicating the successful propagation of the TE mode within the waveguide. After poling under optimized conditions of 100 V for 1.0 h at 60 °C, the *V_π_* of the modulator was determined by measuring the correlation between the intensity of transmitted light and the applied AC voltage. Based on the clear modulation output function depicted in Figure 5b, a *V_π_* of 4.8 V at 1 kHz was measured. The in-device EO coefficient can be calculated using Equation (1) [15]:

(1) $$\gamma_{33}=\frac{\lambda g}{n^{3}\Gamma V_{\pi }L}$$

where λ represents the operation wavelength; *g* is the inter-electrode distance (here, *g* = 6.0 µm); Γ stands for the overlap factor between the optical mode and the applied electric field (Γ = 0.5); and *n* is the effective refractive index of the waveguide. For the in-device EO coefficient, *n*^3^ = *n_eff_*^3^ = 5.83. With an electrode length (*L*) of 1.0 cm, the voltage–length product (*V_π_L*) of the modulator was 4.8 V·cm. The in-device EO coefficient was estimated to be 66 pm/V based on the measured *V_π_L* and the equation employed. It is two times larger than those of the LiNbO_3_ thin films for EO modulators. It clearly indicates that the results confirm the suitability of the low-temperature-processed PZT thin films for EO modulator applications. Although the EO coefficient of low-temperature PZT films is lower than that of high-temperature PZT films, their compatibility with CMOS technology offers a promising pathway for integration into electronic devices, thereby unlocking new avenues for applications.

In Table 1, we compare the electro-optic properties of common materials, including LiNbO_3_ film, BaTiO_3_ film, high-temperature PZT film, and the low-temperature PZT film examined in this experiment. From the table, it is evident that our low-temperature PZT film presents significant advantages in terms of CMOS compatibility.

## 4. Conclusions

In conclusion, we have successfully fabricated well-crystallized PZT thin films on a SiO_2_/Si substrate at a 450 °C annealing temperature. A structural analysis of the films revealed strong preferential (100) growth orientations with a crack-free and smooth surface. Based on the low-temperature PZT films, a MZI waveguide modulator has been designed and fabricated. Notably, the measured *V_π_* is 4.8 V with an electrode length L of 1.0 cm at a wavelength of 1550 nm, yielding an impressive in-device EO coefficient as high as 66 pm/V. The above results unveil the promising EO properties of low-temperature PZT films, representing a notable advancement in the development of PZT-based devices at lower processing temperatures. These findings hold considerable commercial potential for integrated photonics applications.

## Figures and Tables

**Table 1:** The EO properties of commonly used material films.

Material	Processing Temperature	EO Coefficient	CMOS Compatible	Ref.
LiNbO_3_ film	--	31 pm/V	No	[5]
BaTiO_3_ film	660 °C	213 pm/V	No	[24]
High-temperature PZT film	≥600 °C	133 pm/V	No	[15]
Low-temperature PZT film	450 °C	66 pm/V	Yes	This work

## Data Availability

The original contributions presented in this study are included in the article. Further inquiries can be directed to the corresponding author.
